# Spatial Cell Disparity in the Colonial Choanoflagellate *Salpingoeca rosetta*

**DOI:** 10.3389/fcell.2019.00231

**Published:** 2019-10-15

**Authors:** Benjamin Naumann, Pawel Burkhardt

**Affiliations:** ^1^Institute of Zoology and Evolutionary Research, Friedrich Schiller University Jena, Jena, Germany; ^2^Sars International Centre for Marine Molecular Biology, University of Bergen, Bergen, Norway

**Keywords:** choanoflagellate, sponge (Porifera), multicellularity, evolution, collar cells, cell differentiation

## Abstract

Choanoflagellates are the closest unicellular relatives of animals (Metazoa). These tiny protists display complex life histories that include sessile as well as different pelagic stages. Some choanoflagellates have the ability to form colonies as well. Up until recently, these colonies have been described to consist of mostly identical cells showing no spatial cell differentiation, which supported the traditional view that spatial cell differentiation, leading to the co-existence of specific cell types in animals, evolved after the split of the last common ancestor of the Choanoflagellata and Metazoa. The recent discovery of single cells in colonies of the choanoflagellate *Salpingoeca rosetta* that exhibit unique cell morphologies challenges this traditional view. We have now reanalyzed TEM serial sections, aiming to determine the degree of similarity of *S. rosetta* cells within a rosette colony. We investigated cell morphologies and nuclear, mitochondrial, and food vacuole volumes of 40 individual cells from four different *S. rosetta* rosette colonies and compared our findings to sponge choanocytes. Our analysis shows that cells in a choanoflagellate colony differ from each other in respect to cell morphology and content ratios of nuclei, mitochondria, and food vacuoles. Furthermore, cell disparity within *S. rosetta* colonies is slightly higher compared to cell disparity within sponge choanocytes. Moreover, we discovered the presence of plasma membrane contacts between colonial cells in addition to already described intercellular bridges and filo-/pseudopodial contacts. Our findings indicate that the last common ancestor of Choanoflagellata and Metazoa might have possessed plasma membrane contacts and spatial cell disparity during colonial life history stages.

## Introduction

The development from a fertilized egg cell, the so-called zygote, to an embryo made up by hundreds of cells or to a juvenile and adult consisting of more than thousands to billions of cells is a hallmark of animals (Metazoa). Metazoan development is a complex process that is facilitated by the highly coordinated interplay of several not less complex sub-processes such as cell division (cleavage), cell–cell interaction, cell migration, and cell differentiation (Fairclough et al., 2013; Alberts et al., 2014; Brunet and King, 2017). The result of this interplay is a multicellular organism consisting of functionally specialized cells, so-called cell types. Diverse cell types are described in non-bilaterian metazoans such as sponges (Porifera), comb jellies (Ctenophora), *Trichoplax* (Placozoa), and jellyfish (Cnidaria) (Sebé-Pedrós et al., 2018a, b). If these cell types appear in an ontogenetic sequence they are called temporal cell types. Temporal cell types are not restricted to Metazoa, but can also be found in unicellular organisms where cells transition between different cell types during life history (Mikhailov et al., 2009). However, in animals many different cell types are present during the same ontogenetic period. These cell types are then called spatial cell types. In bilaterian metazoans many spatial cell types are highly specialized and sometimes exert only one specific function (Wagner, 2014). In non-bilaterian metazoans spatial cell types are often multifunctional such as epithelial muscle cells in cnidarians, pinacocytes in sponges (both protection, contraction), and the “ocellus” in sponge larvae, a single cell that performs locomotor (steering), photoreceptive, and pigmentation functions (Wagner, 2014).

**FIGURE 1:**
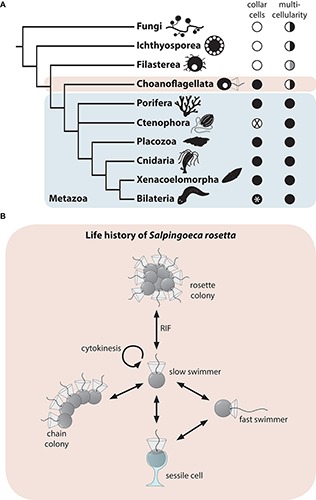
**(A)** Phylogenetic tree showing Choanoflagellata as sister group of the Metazoa (Steenkamp et al., 2005; Carr et al., 2008; Ruiz-Trillo et al., 2008; Adl et al., 2019). In addition, the presence (black circle) and absence (white circle) of collar cells and multicellularity across lineages are shown (Brunet and King, 2017; Laundon et al., 2019). The white asterisk indicates independent secondary losses. Half-filled circles indicate multicellularity in only some species. In Filasterea, multicellularity is achieved by aggregation of single cells (half-filled white-gray circle) instead of clonal division. **(B)** Life history of the choanoflagellate *S. rosetta* after Dayel et al. (2011). RIF, rosette inducing factor (Alegado et al., 2012).

Another multifunctional cell type is the collar cell, a polarized cell with an apical flagellum surrounded by a microvillar collar (Leadbeater, 2015; Arendt et al., 2016; Brunet and King, 2017; Arendt et al., 2019). Collar cells are present in almost all metazoans and their closest relatives, the choanoflagellates (Brunet and King, 2017; Laundon et al., 2019; Figure 1A). The colony-forming choanoflagellate *Salpingoeca rosetta* (Dayel et al., 2011) has emerged as a promising model organism to investigate the evolutionary origin of metazoan multicellularity and cell differentiation (Hoffmeyer and Burkhardt, 2016). Not only is *S. rosetta* easy to culture in the laboratory with a short generation time of 6–8 h and colony induction is highly reproducible, it also has a fully sequenced transcriptome and genome and a suite of functional techniques are now available (Levin et al., 2014; Booth et al., 2018; Wetzel et al., 2018). *S. rosetta* exhibits a complex life history including different temporal cell types during unicellular and colonial life history changes (Dayel et al., 2011; Laundon et al., 2019; Figure 1B). Similar to metazoans, colonies of *S. rosetta* form by mitotic divisions from a single founder cell. Cells within a rosette colony are held together by intercellular cytoplasmatic bridges, filopodia, and an extracellular matrix (Laundon et al., 2019). Rosette colony formation is induced by rosette inducing factor (RIF), a sulfonolipid secreted by the bacterium *Algoriphagus machipongonensis* (Alegado et al., 2012; Woznica et al., 2016).

Whether cells of a rosette colony represent a cluster in which cells are identical to each other or differ from each other is still unclear. Although bulk transcriptomic analyses have shown nearly identical expression patterns for single and colonial cells in *S. rosetta* (Fairclough et al., 2013), a recent study described a distinct morphology of cells in some *S. rosetta* colonies indicating differences of cells within choanoflagellate colonies (Laundon et al., 2019). Understanding whether individual cells of a choanoflagellate colony are identical to each other or different from each other is important for a better understanding of the evolutionary origin of spatial cell differentiation and spatial cell types in the Metazoa. There are several theories on the evolutionary origin of metazoan multicellular development, cell differentiation, and cell types (Fairclough et al., 2010; Sebe-Pedros et al., 2017). As proposed by Haeckel (1874) under the term “Blastaea/Gastraea theory,” animals evolved through “…repeated self-division of [a] primary cell,…” (Haeckel, 1892). In this scenario, the last common ancestor of the Metazoa originated from incomplete cell division of a primary single cell that formed a ball-shaped colony called a “Blastaea” consisting of identical cells (Haeckel, 1874). During evolution, intra-colonial division of labor led to increasing differences between cells resulting in specialized cells representing distinct cell types (King, 2004). In this Blastea hypothesis, spatial cell differentiation evolved before temporal cell differentiation in the stem lineage of the Metazoa (Mikhailov et al., 2009). A hypothesis that contradicts Haeckel’s Blastea hypothesis was proposed by Zakhvatkin (1949). The so-called “Synzoospore theory” claimed that metazoans evolved from a unicellular ancestor that showed a variety of different cell types during different life history stages. According to this theory, temporal cell differentiation was already present and accompanied by spatial cell differentiation in the metazoan stem lineage (Mikhailov et al., 2009).

In this study, we used ultrathin transmission electron microscopy (ssTEM) serial sections of whole rosette colonies of *S. rosetta* to prepare three-dimensional (3D) reconstructions and measure volumes of cell bodies, nuclei, food vacuoles, and mitochondria of 40 individual colonial cells from four colonies. We chose these structures because they can be precisely extracted digitally from the rest of the cellular components at the available resolution (in contrast to other cellular components such as the endoplasmic reticulum, vesicles, the Golgi apparatus, and glycogen granules, etc.). The nuclear and mitochondrial volumes are correlated with cell volume in a variety of unicellular eukaryotes and metazoan cell types (Chan and Marshall, 2010). However, until now such a correlation has not been tested in choanoflagellates. In this context, a deviation of the nuclear volume ratio in some cells could indicate a difference in transcriptional activity in cells within a colony (Chan and Marshall, 2010; Naumova and Dekker, 2010; Jevtić et al., 2014). Food vacuoles on the other hand seem to be more dynamic correlated with food supply rather than cell volume. We therefore expected a lower correlation of this organelle with cell volume. We compared the results with available data (Laundon et al., 2019) on the cellular anatomy of choanocytes of the homoscleromorph sponge *Oscarella carmela* (Ereskovsky et al., 2017). The comparison of cells within a colony and between colonial choanoflagellate cells and sponge choanocytes will help to reveal whether (1) cells in *S. rosetta* rosette colonies are indeed identical (presence of spatial cell disparity), (2) how variable the volume ratios of different cellular organelles are within complete rosette colonies (degree of spatial disparity), and (3) if a similar degree of spatial cell disparity is present in sponge choanocytes (representing a spatially distinct cell type).

## Materials and Methods

### 3D Reconstructions of Complete *S. rosetta* Colonies

A summary of the workflow is shown in Supplementary Figure S1. For our analysis, we used digital image stacks of TEM sections of complete *S. rosetta* colonies (RC1–RC4; *n* = 40 cells), previously published by Laundon et al. (2019) and available from figshare^1^. The image stacks were imported into AMIRA (FEI Visualization Sciences Group) and aligned manually. Subsequently, the cell body and major cell organelles (nucleus, mitochondria, and food vacuoles) were segmented manually. For surface reconstructions, surface models were rendered from the segmented materials, numbers of polygons were reduced and the surfaces were smoothened for the first time. Materials were then imported into Maya (Autodesk), smoothened twice, and colored for final image rendering. For volume renderings, segmented materials were subtracted from the main image stack and exported as separate image stacks. Volume renderings of cells and organelles were prepared using VG Studio Max 2.0 (Volume Graphics).

### Surface Measurements and Volume Calculations

Separated image stacks of cell bodies, nuclei, mitochondria, and food vacuoles of the cells of RC1–RC4 were analyzed with Fiji. Image stacks were imported and masked to create a binary image of the cell body or organelle (black) against a white background. The number of black pixels was counted on each section. The scale bar imprinted in the images was measured in Fiji drawing a line of analogous length. The length of this line in pixels was then divided by the physical length of the scale bar to calculate the physical size for each pixel. All surface area analyses were conducted using unsmoothed, unprocessed materials. Subsequently, surface area measurements were exported to Microsoft Excel 2010 (Microsoft Corporation) and volumes were calculated by multiplying each surface value with the section thickness (RC1: 70 nm; RC2–RC4: 150 nm) and volume ratio calculations and diagrams were prepared.

## Results

### Nuclear Volume Correlates With Cell Size in *S. rosetta* Rosette Colonies

**FIGURE 2:**
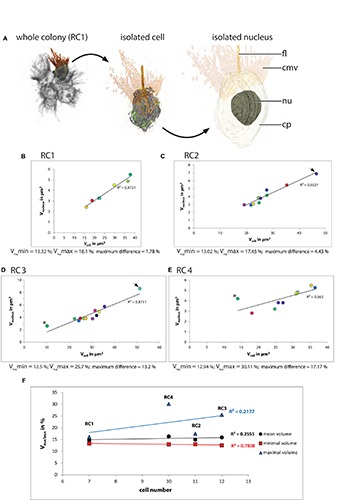
**(A)** 3D-volume-renderings to illustrate the position and size of the nucleus in a colonial *S*. *rosetta* cell. **(B**–**E)** Plots of absolute nuclear volumes against the absolute cellular volume of cells from the four rosette colonies investigated in this study (RC1–RC4). **(F)** Plot of the minimum (red), mean (black) and maximum (blue) relative nuclear volume of each of the four rosette colonies. Cells are color coded according to Table 1. V_nu_max, maximal nuclear volume; V_nu_min, minimal nuclear volume.

In most cells of the four analyzed rosette colonies, the nucleus is located approximately in the middle of the apical–basal axis of the cell (Figure 2A and Supplementary Figures S6–S9). For the relative and absolute volume calculations all sub-structures of the nucleus (the nuclear lamina, eu- and heterochromatin, and the nucleolus) were included (Table 1). A plot of the absolute nuclear volume against the cell volume is shown for each colony in Figure 2.

**TABLE 1:** Absolute and relative nuclear volumes of cells of RC1–RC4.

**RC1**	**C2**	**C1**	**C6**	**C7**	**C3**	**C4**	**C5**					

abs. V_cell_ in μm^3^	15.9781	18.8541	22.1971	22.5861	29.7504	36.4994	37.7110					
abs. V_nu_ in μm^3^	2.4168	3.0355	3.2570	3.2711	4.5026	4.8618	5.4945					
rel. V_nu_ in %	15.13	16.10	14.67	14.48	5.13	13.32	14.57					

**RC2**	**C11**	**C8**	**C2**	**C3**	**C4**	**C7**	**C5**	**C9**	**C6**	**C1**	**C10**	

abs. V_cell_ in μm^3^	19.1235	21.7361	22.5701	24.5022	24.6890	24.7695	25.3476	27.6794	27.7535	35.3241	46.5795	
abs. V_nu_ in μm^3^	2.9657	2.9309	3.0566	3.8402	3.8462	3.2246	3.7845	4.8302	4.1702	5.4659	6.8828	
rel. V_nu_ in %	15.51	13.48	13.54	15.67	15.58	13.02	14.93	17.45	15.03	15.47	14.78	

**RC3**	**C5**	**C6**	**C8**	**C1**	**C4**	**C2**	**C11**	**C12**	**C9**	**C3**	**C10**	**C7**

abs. V_cell_ in μm^3^	10.1994	22.3389	24.2444	24.8660	26.3460	27.3644	30.3141	31.3991	32.1329	33.1629	35.6189	51.2403
abs. V_nu_ in μm^3^	2.6216	3.7827	3.4616	3.8354	3.8246	3.8603	3.7898	5.1014	4.3098	4.8920	5.7396	8.6336
rel. V_nu_ in %	25.70	16.93	14.28	15.42	14.52	14.11	12.50	16.25	13.41	14.75	16.11	16.85

**RC4**	**C5**	**C1**	**C6**	**C10**	**C9**	**C3**	**C7**	**C4**	**C2**	**C8**		

abs. V_cell_ in μm^3^	13.9838	18.0932	24.7512	25.7801	27.1277	30.9752	31.4192	31.4369	35.3603	36.4673		
abs. V_nu_ in μm^3^	4.2099	2.8101	3.2034	3.8192	3.8117	4.6823	4.8305	4.8008	5.4840	5.2734		
rel. V_nu_ in %	30.11	15.53	12.94	14.81	14.05	15.12	15.37	15.27	15.51	14.46		

*abs. V_*cell*_, absolute total cellular volume; abs. V_*nu*_, absolute nuclear volume; rel. V_*nu*_, relative nuclear volume.*

The relative mean nuclear volumes range from 14.77 (RC1) over 14.95% (RC2) and 15.9 (RC3) to 16.32% (RC4). The maximum volume differences between cells within a colony range from 2.78 (RC1) over 4.43% (RC2) and 13.2 (RC3) to 17.17% (RC4). The high maximum volume differences in RC3 and RC4 are mainly due to the large nuclear volume ratios in the carrot-shaped (RC3) and chili-shaped cell (RC4).

In summary, a relatively strong correlation between the nuclear volume and the total cell volume can be recognized (Figures 2B–E). In cells of RC1 (Figure 2B) and RC2 (Figure 2C), the correlation between nuclear volume and total cell volume is strongest. In RC4, the correlation between nuclear and total cell volume is the lowest among the four colonies analyzed. This is again mainly due to the exceptionally high relative nuclear volume in the chili-shaped cell (Figure 2E; black asterisk). The plot of the relative mean, minimal, and maximal nuclear volumes against colony size indicates a higher cell disparity in larger colonies (RC2, RC3, and RC4) compared to RC1 (maximum difference; Figure 2). However, intracolonial cell disparity seems not to increase in a stepwise manner. The minimal and mean relative nuclear volumes do not show a high variation between the colonies, most of the variation comes from the maximal relative nuclear volumes.

### Mitochondrial Volume Correlates With Cell Size in *S. rosetta* Rosette Colonies

**FIGURE 3:**
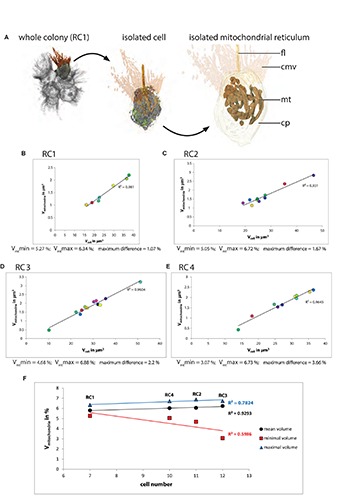
**(A)** 3D-volume-renderings to illustrate the mitochondrial reticulum in a colonial *S. rosetta* cell. **(B–E)** Plots of absolute mitochondrial volumes against the absolute cellular volume of cells from the four rosette colonies investigated in this study (RC1–RC4). **(F)** Plot of the minimum (red), mean (black) and maximum (blue) relative mitochondrial volume of each of the four rosette colonies. Cells are color coded according to Table 2. V_mt_max, maximal mitochondrial volume; V_mt_min, minimal mitochondrial volume.

Most mitochondria in single-cell and colonial *S. rosetta* are organized within a network, called the mitochondrial reticulum, surrounding the nucleus (Leadbeater, 2015; Figure 3A and Supplementary Figures S6–S9). Only the relative and absolute volume of mitochondria located in the cytoplasm of each cell of RC1–RC4 were regarded as functional and reconstructed (Table 2). A plot of the absolute mitochondrial volume against the cell volume is shown for each colony in Figure 3. Mitochondria incorporated into food vacuoles were regarded as non-functional and were not reconstructed.

**TABLE 2:** Absolute and relative mitochondrial volumes of cells of RC1–RC4.

**RC1**	**C2**	**C1**	**C6**	**C7**	**C3**	**C4**	**C5**					

abs. V_cell_ in μm^3^	15.9781	18.8541	22.1971	22.5861	29.7504	36.4994	37.7110					
abs. V_mt_ in μm^3^	1.0130	1.1019	1.1689	1.3063	1.7792	2.0567	2.1986					
rel. V_mt_ in %	6.34	5.84	5.27	5.78	5.98	5.63	5.83					

**RC2**	**C11**	**C8**	**C2**	**C3**	**C4**	**C7**	**C5**	**C9**	**C6**	**C1**	**C10**	

abs. V_*ce*__*ll*_ in μm^3^	19.1235	21.7361	22.5701	24.5022	24.6890	24.7695	25.3476	27.6794	27.7535	35.3241	46.5795	
abs. V_mt_ in μm^3^	1.2810	1.4604	1.1399	1.4480	1.5245	1.4907	1.3743	1.5678	1.7204	2.3561	2.8299	
rel. V_mt_ in %	6.70	6.72	5.05	5.91	6.17	6.02	5.42	5.66	6.20	6.67	6.08	

**RC3**	**C5**	**C6**	**C8**	**C1**	**C4**	**C2**	**C11**	**C12**	**C9**	**C3**	**C10**	**C7**

abs. V_cell_ in μm^3^	10.1994	22.3389	24.2444	24.8660	26.3450	27.3544	30.3141	31.3991	32.1329	33.1629	35.6189	51.2403
abs. V_mt_ in μm^3^	0.4776	1.5074	1.3760	1.6103	1.7979	1.7390	2.0865	2.1444	1.9361	1.9053	2.2667	3.2181
rel. V_mt_ in %	4.58	6.75	5.58	6.48	6.82	6.35	6.88	6.83	6.03	5.75	6.36	6.28

**RC4**	**C5**	**C1**	**C6**	**C10**	**C9**	**C3**	**C7**	**C4**	**C2**	**C8**		

abs. V_ce__ll_ in μm^3^	13.9838	18.0932	24.7512	25.7801	27.1277	30.9752	31.4192	31.4369	35.3603	36.4673		
abs. V_mt_ in μm^3^	0.4290	1.0926	1.6646	1.5366	1.6422	2.0399	1.9526	2.0867	2.2740	2.3652		
rel. V_mt_ in %	3.07	6.04	6.73	5.96	6.05	6.59	6.21	6.64	6.43	6.49		

*abs. V_*cell*_, absolute total cellular volume; abs. V_*mt*_, absolute mitochondrial volume; rel. V_*mt*_, relative mitochondrial volume.*

The relative mean mitochondrial volumes range from 5.81 (RC1) over 6.02% (RC4) and 6.05 (RC2) to 6.24% (RC3). The maximum volume differences between cells within a colony range from 1.09 (RC1) over 1.67% (RC2) and 2.2 (RC3) to 3.66% (RC4). The higher maximum volume differences in RC3 and RC4 are again mainly due to the low mitochondrial volume ratios in the carrot-shaped (RC3) and chili-shaped cells (RC4).

In summary, our data indicate a strong correlation between the mitochondrial volume and the total cell volume in cells of each colony (Figures 3B–E). The relative mean mitochondrial volume increases only slightly with colony size. The relative maximal mitochondrial volume is lowest in RC1 while almost similar in RC2, RC3, and RC4. We observed that in all colonies the majority of the mitochondria of a cell are organized as one large mitochondrial reticulum and only a few solitary mitochondria can be observed. However, an exact measurement of the number of mitochondria was not possible due to the section thickness of 150 nm (in RC2, RC3, and RC4). This thickness in combination with slight distortion artifacts from the sectioning process did not always allow reliable decisions as to whether one mitochondrium is continuous from one section to another or if it ends and another one begins in the following section.

### Food Vacuole Volume Does Not Correlate With Cell Size in *S. rosetta* Rosette Colonies

**FIGURE 4:**
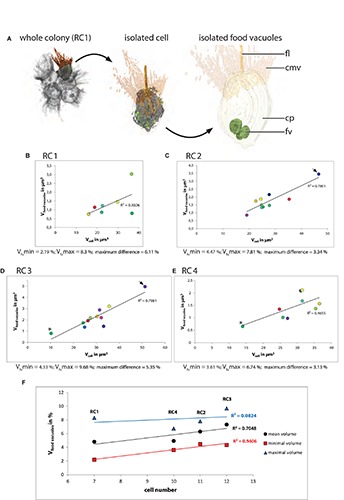
**(A)** 3D-volume-renderings to illustrate the position and size of some food vacuoles in a colonial *S. rosetta* cell. **(B–E)** Plots of absolute food vacuole volumes against the absolute cellular volume of cells from the four rosette colonies investigated in this study (RC1–RC4). **(F)** Plot of the minimum (red), mean (black) and maximum (blue) relative food vacuole volume of each of the four rosette colonies. Cells are color coded according to Table 3. V_fv_max, maximal food vacuole volume; V_fv_min, minimal food vacuole volume.

In most cells food vacuoles are located in the basal half along the apical–basal axis of the cell (Figure 4A and Supplementary Figures S6–S9). In the TEM sections analyzed, food vacuoles appear in different electron densities from high (dark gray) to relatively low (light gray). In between the two “extremes,” food vacuoles appear in different electron densities represented by different shades of gray. The electron density might represent different stages in the digestive cycle. To analyze the complete volume of food vacuoles within a cell we included all recognizable food vacuoles irrespective of their electron density (Table 3). A plot of the absolute food vacuole volume against the cell volume is shown for each colony in Figure 4.

**TABLE 3:** Absolute and relative food vacuole volumes of cells of RC1–RC4.

**RC1**	**C2**	**C1**	**C5**	**C7**	**C3**	**C4**	**C5**				

abs. V_cell_ in μm^3^	15.9781	18.8541	22.1971	22.5861	29.7504	36.4994	36.7110				
abs. V_fv_ in μm^3^	0.7609	1.1468	0.8538	1.2352	1.4518	3.0312	0.8042				
rel. V_fv_ in %	4.76	5.08	3.89	5.47	4.88	8.30	2.19				

**RC2**	**C11**	**C2**	**C3**	**C4**	**C7**	**C5**	**C9**	**C6**	**C1**	**C10**	

abs. V_cell_ in μm^3^	19.1235	22.5701	24.5022	24.6890	24.7695	25.3476	27.6794	27.7535	35.3241	46.5795	
abs. V_fv_ in μm^3^	0.8555	1.7534	1.8748	1.4955	1.3473	1.3772	2.1621	1.4715	1.8635	3.4568	
rel. V_fv_ in %	4.47	7.77	7.65	6.06	5.44	5.43	7.81	5.30	5.28	7.42	

**RC3**	**C5**	**C8**	**C1**	**C4**	**C2**	**C11**	**C12**	**C9**	**C3**	**C10**	**C7**

abs. V_cell_ in μm^3^	10.1994	24.2444	24.8660	26.3460	27.3644	30.3141	31.3991	32.1329	33.1629	35.6189	51.2403
abs. V_fv_ in μm^3^	0.8076	1.7406	1.3623	1.9380	2.1795	2.3129	2.8880	2.2050	1.4348	3.2127	4.9602
rel. V_fv_ in %	7.92	7.18	5.48	7.36	7.96	7.63	9.20	6.86	4.33	9.02	9.68

**RC4**	**C5**	**C6**	**C10**	**C9**	**C3**	**C7**	**C4**	**C2**	**C8**		

abs. V_cell_ in μrn^3^	13.9838	24.7512	25.7801	27.1277	30.9752	31.4192	31.4369	35.3603	36.4673		
abs. V_fv_ in μrn^3^	0.6536	1.3460	1.0135	0.9788	2.0874	2.1149	1.6799	1.3697	1.5665		
rel. V_fv_ in %	4.67	5.44	3.93	3.61	6.74	6.73	5.34	3.87	4.30		

*abs. V_*cell*_, absolute total cellular volume; abs. V_*fv*_, absolute food vacuole volume; rel. V_*fv*_, relative food vacuole volume.*

The relative mean food vacuole volumes range from 4.81 (RC1) over 4.93% (RC4) and 6.29 (RC2) to 7.32% (RC3). The maximum volume differences between cells within a colony range from 3.13 (RC4) over 3.3% (RC2) and 5.35 (RC3) to 6.11% (RC1).

In summary, our data indicate that there is only a weak correlation between food vacuole volume and the total cell volume in cells of each colony (Figures 4B–E). A plot of the relative mean, minimal, and maximal food vacuole volumes against the colony size indicates that the maximum volume difference of food vacuoles is independent from colony size (maximum difference; Figure 4). An exact measurement of the number of food vacuoles was not possible due to the same limitations mentioned for the measurement of the mitochondrial number.

### Cells Within Rosette Colonies of *S. rosetta* Exhibit a Variety of Different Morphologies

**FIGURE 5:**
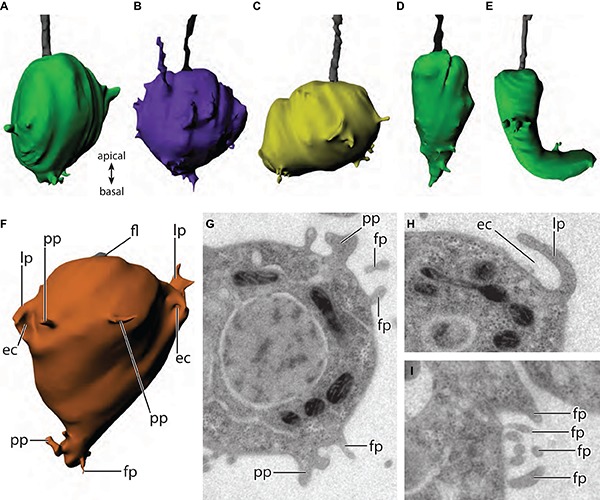
**(A–F)** 3D-surface-reconstructions of cells from four different *S. rosetta* colonies. Cell sizes are not to scale. **(A)** Ovoid morphology (RC1, C6). **(B)** Roundish morphology (RC3, C10). **(C)** Ovoid morphology with the ovoid axis horizontally to the apical–basal axis (RC1, C3). **(D)** “Carrot”-cell (RC3, C5). **(E)** “Chili”-cell (RC4, C5). **(F)** Different types of membrane protrusions within rosette colonies (RC1, C2). **(G–I)** TEM sections of different types of membrane protrusions. ec, endo- or pinocytosis; fl, flagellum; fp, filopodium; lp, lobopodium; pp, pseudopodium.

The individual cells of the four investigated rosette colonies (RC1–RC4) exhibit a variety of volumes/sizes and morphologies (Figure 5). 3D reconstructions of all cells of RC1–RC4 are depicted in Supplementary Figures S2–S5. Many cells exhibit an ovoid morphology, slightly elongated along the apical–basal axis (AB-axis) (Figure 5A). However, some cells exhibit a more roundish (Figure 5B) or ovoid shape horizontally to the AB-axis (Figure 5C). Laundon et al. (2019) described two cells with a distinct morphology within rosette colonies, C5 of RC3 (carrot-like cell; Figure 5D) and C5 of RC4 (chili-like cell; Figure 5E). All cells within a rosette colony exhibit a variety of cell membrane protrusions such as filopodia, pseudopodia, and larger lobopodia-like protrusions that might represent pino- and/or endocytotic events (Figures 5F–I).

In RC1 (seven cells; Supplementary Figure S2), cell volumes range from 15.98 (C2) to 37.71 μm^3^ (C5). Five cells exhibit a more ovoid morphology. Three of these cells (C1, C2, and C6) are slightly elongated along the AB-axis. The other two cells (C3 and C4) are elongated horizontally to the AB-axis. Two cells (C5 and C7) show a more roundish shape.

In RC2 (11 cells; Supplementary Figure S3), cell volumes range from 19.12 (C11) to 46.58 μm^3^ (C10). Nine cells exhibit a more ovoid morphology. Eight of these cells (C1, C2, C4, C5, C6, C7, C9, and C10) are slightly elongated along the AB-axis. C8 is elongated horizontally to the AB-axis. Two cells (C3 and C11) show a more roundish shape.

In RC3 (12 cells; Supplementary Figure S4), cell volumes range from 10.2 (C5) to 51.24 μm^3^ (C7). Nine cells exhibit a more ovoid morphology. Eight of these cells (C1, C3, C5, C6, C7, C8, C9, C11, and C12) are slightly elongated along the AB-axis. As previously reported, C5 exhibits a distinct slender, carrot-shaped morphology (Laundon et al., 2019). C4 is elongated horizontally to the AB-axis. Two cells (C2 and C10) are more roundish. C7, the largest cell of the colony, and C12 shows an exceptional high number of membrane protrusions (Supplementary Figure S4).

In RC4 (10 cells; Supplementary Figure S5), cell volumes range from 13.98 (C5) to 36.47 μm^3^ (C8). Seven cells (C1, C3, C4, C6, C7, C9, and C10) exhibit a more ovoid morphology, slightly elongated along the AB-axis. As previously reported, C5 exhibits a distinct slender, chili-shaped morphology (Laundon et al., 2019). Two cells (C2 and C8) show a more roundish shape.

**FIGURE 6:**
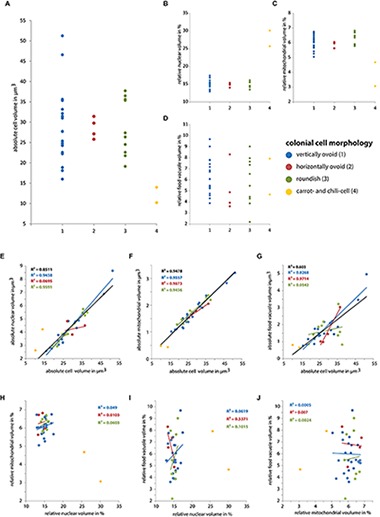
**(A–D)** Plots of the different types of cell morphology of colonial *S. rosetta* cells against cellular volume and relative organelle volumes. **(E–J)** Plots of the absolute **(E–G)** and relative organelle volumes **(H–J)** against each other. A regression line is shown in the same color as it corresponding type of cell morphology. The black regression line in panels **E–G** is calculated from all 40 cells.

To determine if specific cell morphologies correspond to specific nuclear, mitochondrial, and food vacuoles volumes we plotted cell morphologies against the total cellular volume and volumes of the investigated organelles (Figure 6). No profound differences of total cell volume, nuclear, mitochondrial, and food vacuole volume were found except for the carrot-shaped and chili-shaped cells (Figures 6A–D). Subsequently, we plotted the relative total cellular, nuclear, mitochondrial, and food vacuole volumes against each other to test if there are specific patterns for the distinct cell morphologies (Figures 6E–J). There is a strong correlation of the nuclear and mitochondrial volumes with the total cell volume. This confirms our earlier observations where the four colonies were considered separately. The horizontally ovoid cells however exhibit a lower correlation of nuclear volume to cell volume compared to the other cell morphologies. Regarding the food vacuole to cell volume ratio, the overall correlation was much lower compared to nuclear and mitochondrial volumes. The lowest correlation of food vacuole to cell volume ratio can be found in roundish cells (Figure 6G). No correlations were found when nuclear, mitochondrial, and food vacuole ratios were plotted against each other (Figures 6H–J).

### Quantitative Analysis of Cell–Cell Contacts Reveals Plasma Membrane Contacts in Colonial Cells of *S. rosetta*

**FIGURE 7:**
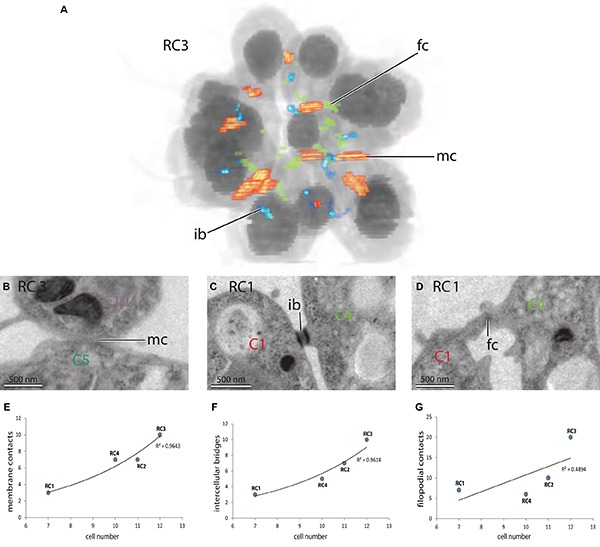
**(A)** 3D-volume-rendering of *S. rosetta* rosette colony RC3 to illustrate the distribution of cell–cell contacts within a colony. **(B–D)** TEM sections of different colonies highlight various types of cell–cell contacts in colonial *S. rosetta* cells. **(B)** Plasma membrane contact between C5 and C10 (RC3). **(C)** Intercellular bridge between C1 and C4 (RC1). **(D)** Filopodial contact between C1 and C4 (RC1). **(E–G)** Plots of the number of specific cell–cell contacts against the colony size (measured in cell number). ib, intercellular bridge; fc, filopodial contact; mc, membrane contact.

Numerous intercellular bridges and filo-/pseudopodial contacts can be found between cells in a colony (Figure 7; Leadbeater, 2015; Laundon et al., 2019). Additionally to our best knowledge, we report plasma membrane contacts between some cells of a colony for the first time (Figures 7A,B). These membrane contacts are found in all four colonies and range from relatively small areas (around 100 nm length on a section) up to areas of a length of >500 nm (length on a section).

We quantified the number of the newly found plasma membrane contacts in the colonies used in this study. It seems that the number of plasma membrane contacts increases with the colony size (Figure 7E). This is similar to the number of intercellular bridges (Figure 6F; Laundon et al., 2019). The number of filopodial/pseudopodial contacts between cells within the colonies seems not correlated with colony size (Figure 7G). A detailed summary of the types (intercellular bridges, membrane contact, and filopodial contact) and number of connections of individual cell of each of the four investigated colonies is given in Table 4.

**TABLE 4:** Types of cell–cell contacts of cells of RC1-RC4.

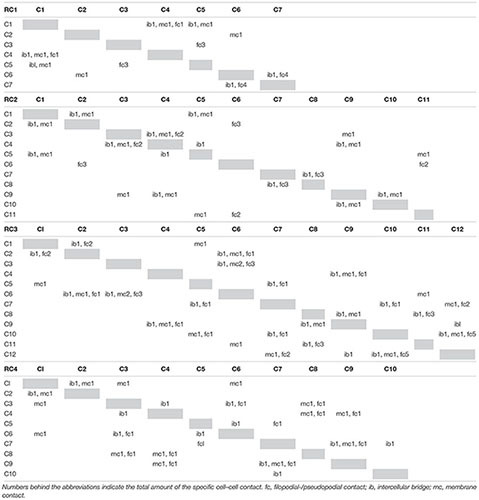

## Discussion

**FIGURE 8:**
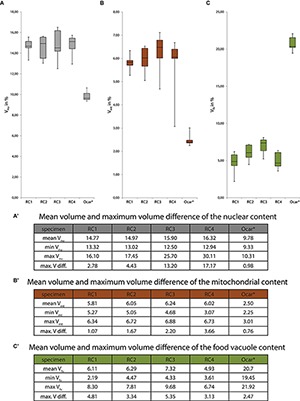
**(A–C)** Box and whisker plots of the relative volumes of different cell organelles of *S. rosetta* colonies (RC1–RC4) and five choanocytes of *O. carmela* (Ocar). Values in the tables are given in % in relation to the total cellular volume. Asterisk, data taken from Laundon et al. (2019). **(A)** Nuclear volumes (gray box). **(B)** Mitochondrial volumes (brown box). **(C)** Food vacuole volumes (green box). **(A’–C’)** Tables showing the minimum, maximum, and mean volumes as well as the maximum volume differences for each of the investigated organelles. max. V diff., maximum volume difference; mean V, mean volume; Vmax, maximal volume; Vmin, minimal volume.

In this study, we analyzed cell morphologies, volumes of cell bodies, and volumes of some major organelles (nucleus, mitochondria, and food vacuoles) of four *S. rosetta* rosette colonies (40 cells in total). The aims were: (1) To investigate whether cells in rosette colonies of *S. rosetta* are indeed identical or if they differ from each other. (2) In case they differ from each other, to what degree do they vary in terms of morphology, cell volume, and organelle content? (3) To compare the intracolonial cell differences to the differences within a group of choanocytes of the homoscleromorph sponge *O. carmela*. The differences of cells within a colony are here described in a relative way using the term “cell disparity” (indicated by maximum volume differences in this study). Identical cells show no disparity at all, the maximum volume difference within a colony/tissue is zero. In contrast, cells that exhibit maximum volume differences lead to a certain degree of cell disparity within a colony/tissue (Figure 8).

### Rosette Colonies of *S. rosetta* Exhibit Spatial Cell Disparity Regarding Cell Morphology and the Nuclear and Mitochondrial Content

Cells within *S. rosetta* colonies are not identical but show spatial cell disparity regarding their nuclear, mitochondrial, and food vacuole contents (Figure 8). The largest difference of the nuclear and mitochondrial contents is found in the carrot-shaped (RC3) and chili-shaped cells (RC4). If these two cells are removed from the analysis the overall cell disparity declines in these two colonies (RC3: V_nu_ from 13.2 to 4.43%, V_mt_ from 2.2 to 1.1%; RC4: V_nu_ from 17.17 to 2.59%, V_mt_ from 3.66 to 0.77%). In this regard the carrot-shaped and the chili-shaped cells are the main reason for the cell disparity observed in RC3 and RC4. However, since it is not known whether the carrot- and chili-shaped cells exert special functions within a colony it cannot be excluded that other cells within the colony would compensate for the removal of these cells by internal organelle changes. Due to the lack of knowledge if there is a functional role of the carrot-shaped and chili-shaped cells we consider all cells integrated within a colony and discuss our results with the carrot-shaped and chili-shaped cell included in the analysis.

The maximum volume differences of the nuclei (Figure 8A) could indicate slightly different transcription activities of cells within a colony. It is known that changes in nuclear size and form can be the cause or result of changes in chromatin organization, gene expression, and other physiological processes of the cell (Naumova and Dekker, 2010; Jevtić et al., 2014). A study on myotube formation in human myoblasts has shown that a decrease in nuclear size is correlated with altered histone modifications, chromatin remodeling, and gene silencing (Rozwadowska et al., 2013). The comparison of nuclear volumes of colonial cells (this study) with single cells of *S. rosetta* (Laundon et al., 2019) shows no difference between the two life history stages. Therefore, the observed differences of the relative nuclear volumes (e.g., in the carrot-shaped and chili-shaped cells or the horizontally ovoid cells) might be due to asynchronous cell-cycles, different metabolic states, or even different intra-colonial oxygen levels affecting transcriptional activity (Zhou et al., 2007) rather than different cell types within cells of a colony. The big difference of the relative nuclear volumes of the carrot-shaped and chili-shaped cell could result from a high asynchrony in cell-cycle/metabolism compared to the other cells in the colony or, in this special case, indicate more specialized function of these two cells.

Maximum volume differences, resulting in spatial cell disparity, were also observed for the relative mitochondrial (Figure 8B) and food vacuole volumes (Figure 8C). Laundon et al. (2019) suggested that there is a significant difference in mitochondrial number (single cells: 25.3 ± 5.8 vs. colonial cells: 4.3 ± 4.2) but not mitochondrial volume (single cells: 5.08 ± 1.14% vs. colonial cells: 6.63 ± 0.42%) between single and colonial *S. rosetta* cells. This difference in number could be due to a higher demand on energy necessary for locomotion in single-cell *S. rosetta*. We confirm the results from Laundon et al. (2019) regarding the relative mean volume of the mitochondrial reticulum in colonial *S. rosetta* (RC1: 5.81%; RC2: 6.05%; RC3: 6.24%; RC4: 6.02%; Figure 8B). Our reconstructions of the mitochondrial reticulum of colonial cells also support the presence of a lower number of mitochondria in colonial cells (Laundon et al., 2019). However, it was not possible to determine the exact number of mitochondria in cells of RC2, RC3, and RC4 due to the thickness (150 nm) of the sections. The lower mitochondrial number in colonial cells could be due to mitochondrial fusion. It is known that mitochondrial fusion is stimulated by energy demand and stress while fission may generate new organelles and facilitates quality control (Youle and Van Der Bliek, 2012). Limited mitochondrial fusion results in improper embryogenesis and is associated with some human diseases (Chen and Chan, 2010). Therefore, mitochondrial fusion might act as a “defense mechanism” against cellular aging (Westermann, 2002). Similar to our speculation on the variety of cell morphologies, the absence of extensive directed locomotion and the decreasing demand for high energy consumption in colonies might release cells from the constraint of having a high number of active, ATP-producing mitochondria. This may allow for a higher degree of mitochondrial fusion and increased longevity of mitochondrial function (Chen and Chan, 2010). The food vacuoles are regarded as the most dynamic organelle type investigated in this study. The observed maximum volume differences of the food vacuole content of cells within colonies range from 3.13 to 5.35%. This difference might be most likely due to different metabolic rates and differences in the food uptake of individual cells.

Colonies show a variety of cell morphologies from roundish over ovoid (along the AB-axis or horizontal to the AB-axis) to two extreme morphologies, the carrot-shaped (RC3; Laundon et al., 2019) and the chili-shaped cell (RC4; Laundon et al., 2019).

We interpret our results that cells within a colony exhibit spatial cell disparity most likely on the basis of asynchronous cell cycles and different metabolic rates. The carrot-shaped and chili-shaped cells with their larger nuclear and lower mitochondrial content might already exert specialized functions compared to other cells within the same colony.

### Rosette Colonies of *S. rosetta* Exhibit Slightly Higher Spatial Cell Disparity Compared to Sponge Choanocytes

**FIGURE 9:**
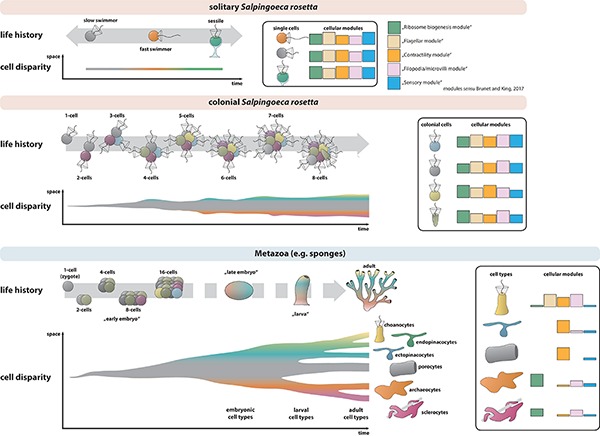
Hypothesis on spatial and temporal cell disparity in *S. rosetta* single cells, colonies, and metazoans (e.g., sponges). Three cell types are described in solitary life history stages of *S. rosetta* (Dayel et al., 2011). Each of the solitary cell types might exhibit distinct expression levels of several constitutive cellular modules (*sensu* Brunet and King, 2017) and cell disparity varies only in time but not in space. In colonial *S. rosetta*, cell disparity additionally varies in space. Upon increase of colony size the probability in cell disparity increases. In metazoans, cell number increases tremendously leading to a high degree of cell disparity. During development (and life history), cells differentiate into distinct cell types that express a specific set of cellular modules. This process decreases cell disparity between cells of the same cell types but increases cell disparity between cells belonging to different cell types.

A comparison of the nuclear volume data of colonial *S. rosetta* cells with data from choanocytes of the homoscleromorph sponge *O. carmel*a (Laundon et al., 2019) shows that the mean relative nuclear volume of *O. carmela* choanocytes (9.78%, *n* = 5; Figure 7A) is around one-third lower as the mean nuclear volume in cells of *S. rosetta* colonies (e.g., RC1: 14.77%, *n* = 7; Figure 8A). Additionally, the maximum volume difference of the nuclear volume of *O. carmela* choanocytes (within the same choanocyte chamber of a sponge individual) is almost three times lower as the smallest maximum volume difference within one of the analyzed *S. rosetta* colonies (e.g., RC1: maximum volume difference = 2.78%; Figure 8A). These differences in the relative nuclear volume can be explained in two ways. The first explanation focuses on alterations of the nuclear volume due to cell division. For example, in the demosponge *Hymeniacidon sinapium*, choanocytes divide every 20–40 h (Shore, 1971). It is interesting to note here that archaeocytes of another demosponge, *Amphimedon queenslandica*, differentiate into choanocytes within only 2 h without prior cell division (Sogabe et al., 2019). Cells of *S. rosetta* rosette colonies in contrast divide every 6–8 h (Fairclough et al., 2010). Due to the shorter cell cycle length of choanoflagellates compared to sponges it might be that the fixed *S. rosetta* colonies contained, by chance, more cells in the G2-phase of the cell cycle. G2-phase nuclei are often larger than nuclei during for example the G1-phase due to the doubling of the DNA during the S-phase preceding the G2-phase (Maeshima et al., 2011). The second explanation focuses on the specialized function of sponge choanocytes. Colonial choanoflagellate cells have been regarded as being more or less similar meaning that they show no real division of labor as present in Metazoa (Leadbeater, 2015). Therefore choanoflagellate cells have to be “all-rounder” and constitutively express a variety of cellular modules such as for instance a ribosome biogenesis module, a flagellar module, a contractility module, and a filopodia/microvilli module (Brunet and King, 2017) to encounter all possible functional demands. Choanocytes in contrast, existing in a multicellular organism with other cell types, are specialized on only a few functions such as creating water current and food uptake (Simpson, 2012; Mah et al., 2014; Dunn et al., 2015). Therefore, sponge choanocytes may not express a multitude of different cellular modules. Instead they might express only some modules in a cell type specific manner (Brunet and King, 2017; Figure 9). The expression of fewer cellular modules could be reflected by a decreased number of active genes and higher values of densely packed heterochromatin resulting in a smaller relative nuclear volume. Specialization could also explain the lower cell disparity in *O. carmela* choanocytes compared to colonial cells of *S. rosetta*. These arguments can be tested by investigating the chromatin architecture and euchromatin/heterochromatin ratios in “all-rounder” colonial cells of *S. rosetta* and specialized choanocytes of *O. carmela*.

The view of choanoflagellate cells as “all-rounders” and sponge choanocytes as functional specialists is further supported by the two times higher mean mitochondrial volume and the almost four times lower mean food vacuole volume of colonial *S. rosetta* cells compared to *O. carmela* choanocytes (Figure 8B). As mentioned by Laundon et al. (2019) choanocytes as specialized cells without locomotory function might not need such high amounts of energy as free swimming *S. rosetta* cells with dual functions (food acquisition and locomotion) do. Additionally, we find that the maximum volume difference of the mitochondrial volumes of cells within *S. rosetta* colonies is around 1.5–4.8 times higher compared to the analyzed *O. carmela* choanocytes of the same individual (Figure 8B). The cell disparity in choanoflagellate colonies could indicate early stages of “division of labor” (Bonner, 2009). Cells of a colony are connected by intracellular bridges, pseudo-/filopodia (Dayel et al., 2011; Leadbeater, 2015; Laundon et al., 2019) and membrane contacts (this study). Some of these structures could serve in exchange of metabolic compounds and could explain that certain cells within a colony must reduce the cellular volume devoted to mitochondria and increase the expression of other “cellular modules” (*sensu* Brunet and King, 2017) while others increase the mitochondrial volume to cover the total energetic demands of the colony. Of special interest would be a comparison of the mitochondrial and food vacuole volumes between sessile, thecate *S. rosetta* and sponge choanocytes. If the “loss of constraints” hypothesis is correct, sessile *S. rosetta* should exhibit higher volumes of food vacuole and lower mitochondrial volumes than “slow and fast swimmer” cells and therefore be more similar to choanocytes.

### Plasma Membrane Contacts in *S. rosetta* Rosette Colonies

Cell–cell contacts and differential cell adhesion are key features during development and morphogenesis of any metazoan embryos (Gilbert, 2013). These contacts can be established in different ways utilizing intercellular bridges, filo-/pseudopodia and/or whole areas of the cell membrane. Intercellular bridges have been described in *S. rosetta* and several colony-forming choanoflagellates (Karpov and Coupe, 1998; Dayel et al., 2011; Leadbeater, 2015; Laundon et al., 2019). They have been hypothesized to function as channels for intercellular communication (Fairclough et al., 2013). It has been shown that the number of intercellular bridges increases with the size of *S. rosetta* colonies (Laundon et al., 2019). In this study we report the presence of plasma membrane contacts between some cells of rosette colonies of *S. rosetta*. Our data show that the total number of intercellular plasma membrane contacts is comparable to the number of intercellular bridges and increases with colony size in a very similar pattern as observed for intercellular bridges (Figures 7A,B,D,E). It is thought that Cadherins are key mediators of plasma membrane contacts and cell adhesion in metazoans (King et al., 2003; Cereijido et al., 2004; Halbleib and Nelson, 2006). Twenty-three cadherins have been found in the strictly solitary choanoflagellate *Monosiga brevicollis.* Two of these cadherins localize in the microvillar collar and colocalize with the actin cytoskeleton (Abedin and King, 2008). In *S. rosetta*, 29 proteins containing cadherin domains have been described (Nichols et al., 2012). However, the functions of these *S. rosetta* cadherins are still unknown (Fairclough et al., 2013). Some of the cadherins are differentially expressed during different stages of *S. rosetta* life history. Interestingly, two of these cadherins (PTSG_06458 and PTSG_06068) are upregulated in colonies compared to single cells (Fairclough et al., 2013). Further investigation of the spatial expression patterns of these two and other cadherins are crucial to clarify the properties and potential functions of intercellular membrane contacts in colonial choanoflagellates. In contrast to intercellular bridges and membrane contacts, the number of filo-/pseudopodial cell–cell contacts seems not tightly correlated with the size of a colony and might be a more variable and transient type of cell–cell contacts (Figures 7C,F). It remains to be examined if some more stable types of choanoflagellate cell–cell contacts (intercellular bridges, plasma membrane contacts) have homologous structures in metazoans and therefore might have been present in the last common ancestor of choanoflagellates and metazoans.

### Spatial Cell Disparity and the Last Common Ancestor of Choanoflagellates and Metazoans

Our study reports spatial cell disparity within rosette colonies of the choanoflagellate *S. rosetta*. The major part of this spatial cell disparity might be due to asynchronous cell-cycles (nuclear and cell volumes) and variations in metabolic processes (mitochondrial and food vacuole volumes). Single choanoflagellate cells for example may only exhibit cell disparity in time (life history) but not in space because the same single cell can only have one specific identity at the time (Figure 9). A choanoflagellate colony consisting of several cells can additionally exhibit cell disparity in space since different cells can have different identities. In theory, upon increase of cell numbers in a colony, increased cell identities can be present at the same time point leading to a higher possible cell disparity within the colony (Figure 9). However, a generalization of the idea that cell disparity increases with colony size is limited by the sample size investigated in this study. More *S. rosetta* colonies must be investigated in detail to test this idea. Another aim was (3) to compare our data to previously published data on nuclear, mitochondrial, and food vacuole volumes in choanocytes of the homoscleromorph sponge *O. carmela* (Laundon et al., 2019). *O. carmela* choanocytes exhibit smaller nuclear and mitochondrial but larger food vacuole volumes compared to cells of *S. rosetta* colonies (Laundon et al., 2019, this study; Figure 8). Additionally to these findings, we showed that the maximum volume difference of each of the three organelle volumes is lower compared to colonial choanoflagellate cells (Figure 8). It seems that sponge choanocytes are not only more specialized on food acquisition (high volume of food vacuoles and lower mitochondrial and nuclear volumes) but also more similar to each other than individual cells in a colony of *S. rosetta*. Therefore, choanocytes seem to exhibit lower spatial cell disparity compared to colonial *S. rosetta* cells (Figure 9). Is it possible to integrate this idea into an evolutionary context to explain the origin of metazoan cell types?

In contrast to the “Blastea/Gastrea” theory (Haeckel, 1874, 1892), the Synzoospore hypothesis proposed that the origin of the Metazoa corresponds to the transition from temporal to spatial cell differentiation (Zakhvatkin, 1949; Mikhailov et al., 2009). Zakhvatkin (1949) suggested that the last common ancestor of the Metazoa might have been an organism that already exhibited different cell types during different life history phases (temporal cell disparity and cell differentiation) as it can be seen in many protozoan taxa such as *S. rosetta* (Mikhailov et al., 2009; Dayel et al., 2011). During evolution, this organism acquired a benthic colonial or multicellular phase that was made up by cells of different cell types already present in the single cell stages of the life history of this organism. Mikhailov et al. (2009) suggested that it is unlikely that genetic programs of cell differentiation evolved *de novo* in this last common ancestor of the Metazoa. Instead, pre-existing mechanisms (cell differentiation programs) were used to integrate the different cell types that already occur during single cell life history phases of this organism. On the basis of a detailed ultra-structural study, Laundon et al. (2019) suggested that colonial cells of *S. rosetta* might represent a distinct cell type instead of a conglomerate of identical “slow swimmer” cells. The carrot-shaped and chili-shaped cell may also represent distinct cell types (Laundon et al., 2019), which is supported by our finding of high cell disparity in *S. rosetta* colonies.

Despite the controversy whether metazoans evolved from an ancestor exhibiting a “simple” or more complex life history, two main advantages have been proposed to drive positive selection for multicellularity in general. The first is an increase of size (Bonner, 2009). Larger organisms/colonies might experience a lower predation pressure compared to smaller organisms/colonies (intercolonial competition) (Bonner, 2009). However, after a certain size has been reached, cells within a colony might exhibit competition for space and nutrient availability (intracolonial competition). Therefore, selection might favor a better integration of cells by colonizing different “niches,” gradually becoming more different from each other (increasing spatial cell disparity) and eventually are recognized as different cell types. The result might have been a multicellular organism with different cell types that exhibit division of labor (Bonner, 2009).

Recently it was shown that sponge archeocytes, and not sponge choanocytes, share similar gene expression profiles with choanoflagellates (Sogabe et al., 2019), thus questioning the close evolutionary relationship of choanoflagellates and choanocytes. In addition, sponge archaeocytes can differentiate into many other sponge cell types, including choanocytes and the authors suggested an alternative path to the first animals (Sogabe et al., 2019). Not a cell with a choanoflagellate morphology was the ancestral cell that preceded animal multicellularity, but a cell which was able to differentiate quickly into different cell types, similar to a stem cell found in many different animals. Our data now hint at the possibility that also choanoflagellate cells have the capacity for differentiation. To further our understanding on the evolutionary origin of animal cell types and cell differentiation 3D reconstructions and detailed volumetric analyses of additional choanoflagellate cells and additional sponge choanocytes and archeocytes are needed.

## Data Availability Statement

The datasets generated for this study are available on request to the corresponding author.

## Author Contributions

BN and PB designed the study and wrote the manuscript. BN performed the experiments and analyzed the data.

## Conflict of Interest

The authors declare that the research was conducted in the absence of any commercial or financial relationships that could be construed as a potential conflict of interest.

## Footnotes

1 10.6084/m9.figshare.7346750.v2

## Supplementary Material

FIGURE S1 Scheme of the workflow and software types used in this study.

FIGURE S2 3D-surface-renderings of cells of a rosette colony of *S. rosetta* (RC1). Cells are not to scale. **(A)** 3D-view of the whole colony from different angles. The color spectrum indicates the identity of the different cells. **(B–H)** Single views of cells of the colony. Cells are oriented along the apical (flagellar)–basal axis. The volume of the whole cell body is given beneath every cell.

FIGURE S3 3D-surface-renderings of cells of a rosette colony of *S. rosetta* (RC2). Cells are not to scale. **(A)** 3D-view of the whole colony from different angles. The color spectrum indicates the identity of the different cells. **(B–L)** Single views of cells of the colony. Cells are oriented along the apical (flagellar)–basal axis. The volume of the whole cell body is given beneath every cell. C10 was not completely sectioned and half of the volume was added to estimate the total cellular volume indicated by the dotted line.

FIGURE S4 3D-surface-renderings of cells of a rosette colony of *S. rosetta* (RC3). Cells are not to scale. **(A)** 3D-view of the whole colony from different angles. The color spectrum indicates the identity of the different cells. **(B–M)** Single views of cells of the colony. Cells are oriented along the apical (flagellar)–basal axis. The volume of the whole cell body is given beneath every cell.

FIGURE S5 3D-surface-renderings of cells of a rosette colony of *S. rosetta* (RC4). Cells are not to scale. **(A)** 3D-view of the whole colony from different angles. The color spectrum indicates the identity of the different cells. **(B–K)** Single views of cells of the colony. Cells are oriented along the apical (flagellar)–basal axis. The volume of the whole cell body is given beneath every cell.

FIGURE S6 3D volume renderings of the nucleus, mitochondrial reticulum, and food vacuoles of cells of a rosette colony of *S. rosetta* (RC1). **(A–G)** Cell one (C1) to cell seven (C7). Cells are not to scale. Cells are oriented along the apical (flagellar)–basal axis. The cell body is shown half transparent. The nucleus is colored in dark gray and the mitochondrial reticulum in brown. Food vacuoles with high electron density are colored in light green while food vacuoles with lower electron density are colored in dark gray.

FIGURE S7 3D volume renderings of the nucleus, mitochondrial reticulum, and food vacuoles of cells of a rosette colony of *S. rosetta* (RC2). **(A–K)** Cell one (C1) to cell 11 (C11). Cells are not to scale. Cells are oriented along the apical (flagellar)–basal axis. The cell body is shown half transparent. The nucleus is colored in dark gray and the mitochondrial reticulum in brown. Food vacuoles with high electron density are colored in light green while food vacuoles with lower electron density are colored in dark gray.

FIGURE S8 3D volume renderings of the nucleus, mitochondrial reticulum, and food vacuoles of cells of a rosette colony of *S. rosetta* (RC3). **(A–L)** Cell one (C1) to cell 12 (C12). Cells are not to scale. Cells are oriented along the apical (flagellar)–basal axis. The cell body is shown half transparent. The nucleus is colored in dark gray and the mitochondrial reticulum in brown. Food vacuoles with high electron density are colored in light green while food vacuoles with lower electron density are colored in dark gray.

FIGURE S9 3D volume renderings of the nucleus, mitochondrial reticulum, and food vacuoles of cells of a rosette colony of *S. rosetta* (RC4). **(A–J)** Cell one (C1) to cell ten (C10). Cells are not to scale. Cells are oriented along the apical (flagellar)–basal axis. The cell body is shown half transparent. The nucleus is colored in dark gray and the mitochondrial reticulum in brown. Food vacuoles with high electron density are colored in light green while food vacuoles with lower electron density are colored in dark gray.
