# Supplementary material for: Spatial Cell Disparity in the Colonial Choanoflagellate Salpingoeca rosetta
Source: Front Cell Dev Biol. 2019 Oct 15;7:231. doi: 10.3389/fcell.2019.00231 (PMC6803389; doi:10.3389/fcell.2019.00231)
Supplement: FIGURE S1 — Scheme of the workflow and software types used in this study. [file Image_1.pdf]

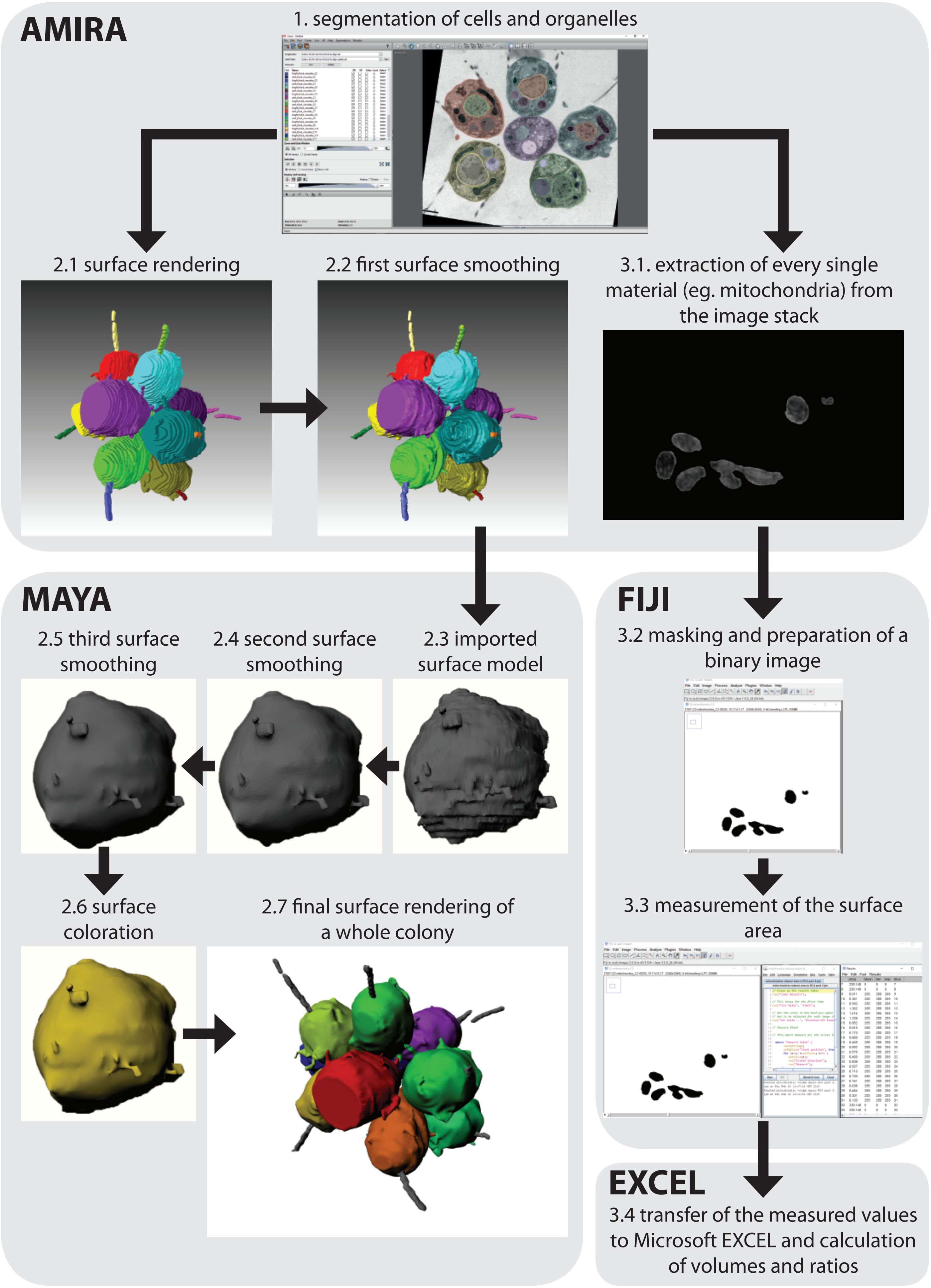


Suppl. Figure 1
