# Supplementary figures and images for: Spatial Cell Disparity in the Colonial Choanoflagellate Salpingoeca rosetta
Source: Front Cell Dev Biol. 2019 Oct 15;7:231. doi: 10.3389/fcell.2019.00231 (PMC6803389; doi:10.3389/fcell.2019.00231)

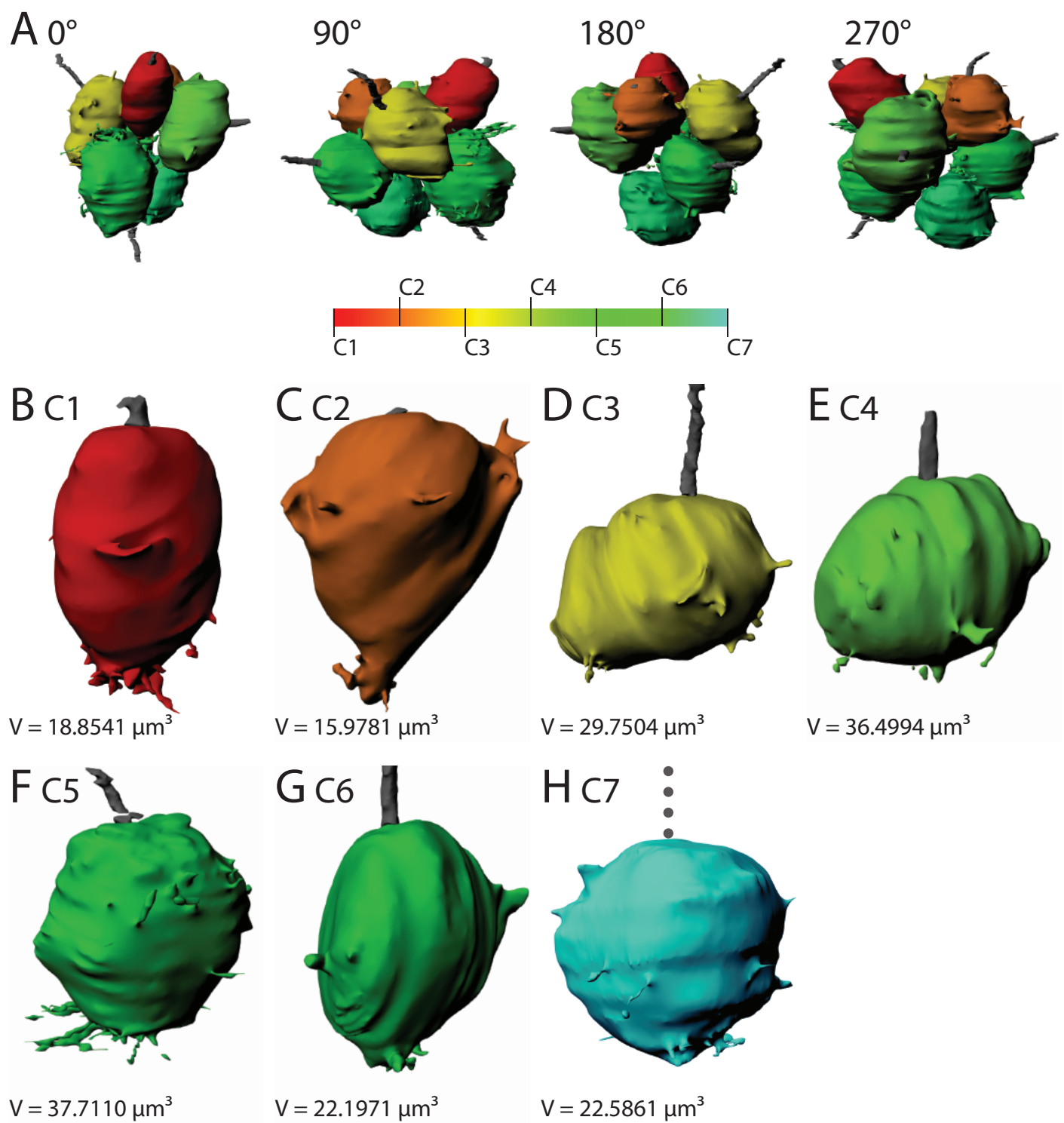

Suppl. Figure 2

Supplement: FIGURE S2 — 3D-surface-renderings of cells of a rosette colony of S. rosetta (RC1). Cells are not to scale. (A) 3D-view of the whole colony from different angles. The color spectrum indicates the identity of the different cells. (B–H) Single views of cells of the colony. Cells are oriented along the apical (flagellar)–basal axis. The volume of the whole cell body is given beneath every cell. [file Image_2.pdf]

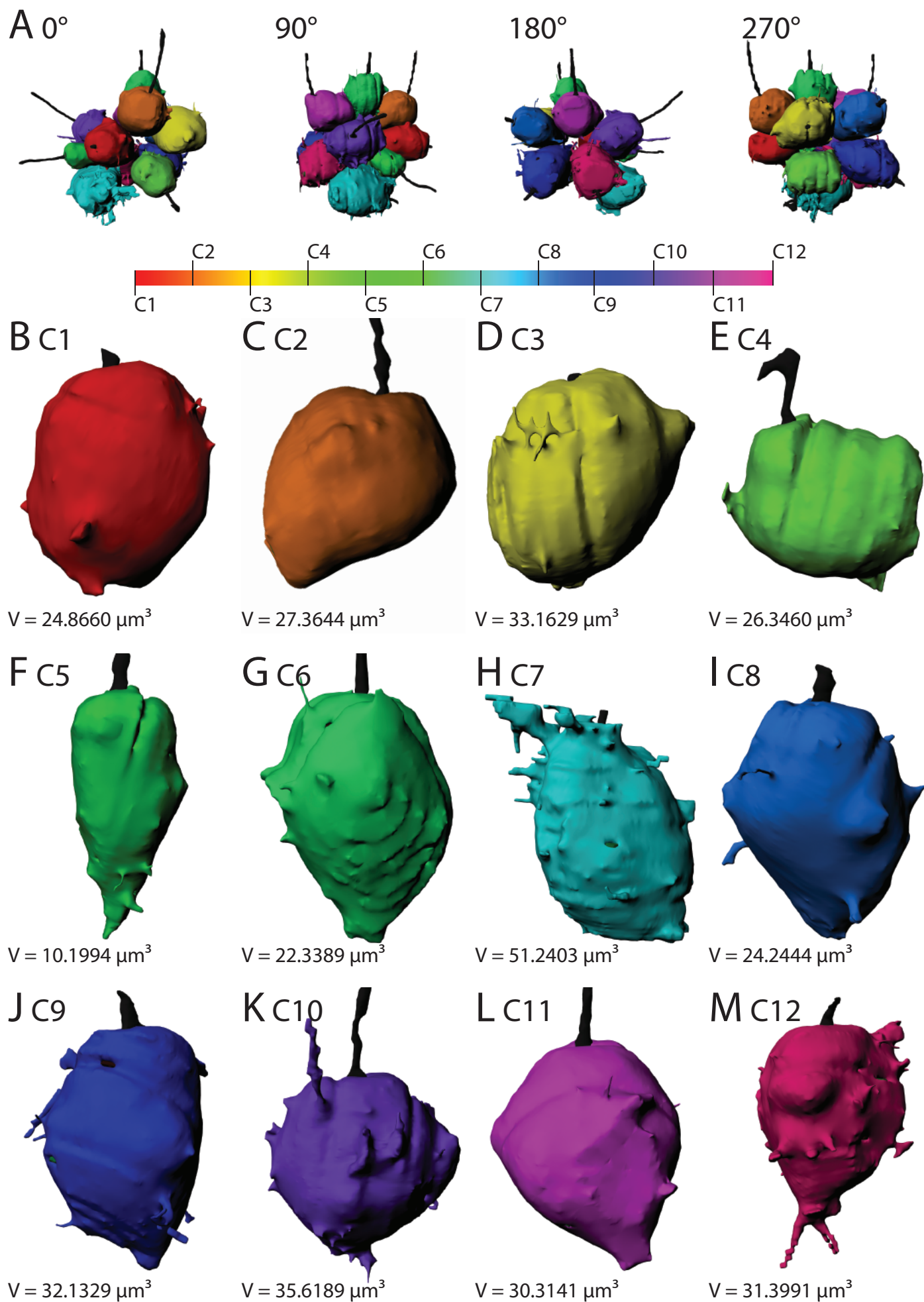

Suppl. Figure 4

Supplement: FIGURE S4 — 3D-surface-renderings of cells of a rosette colony of S. rosetta (RC3). Cells are not to scale. (A) 3D-view of the whole colony from different angles. The color spectrum indicates the identity of the different cells. (B–M) Single views of cells of the colony. Cells are oriented along the apical (flagellar)–basal axis. The volume of the whole cell body is given beneath every cell. [file Image_4.pdf]

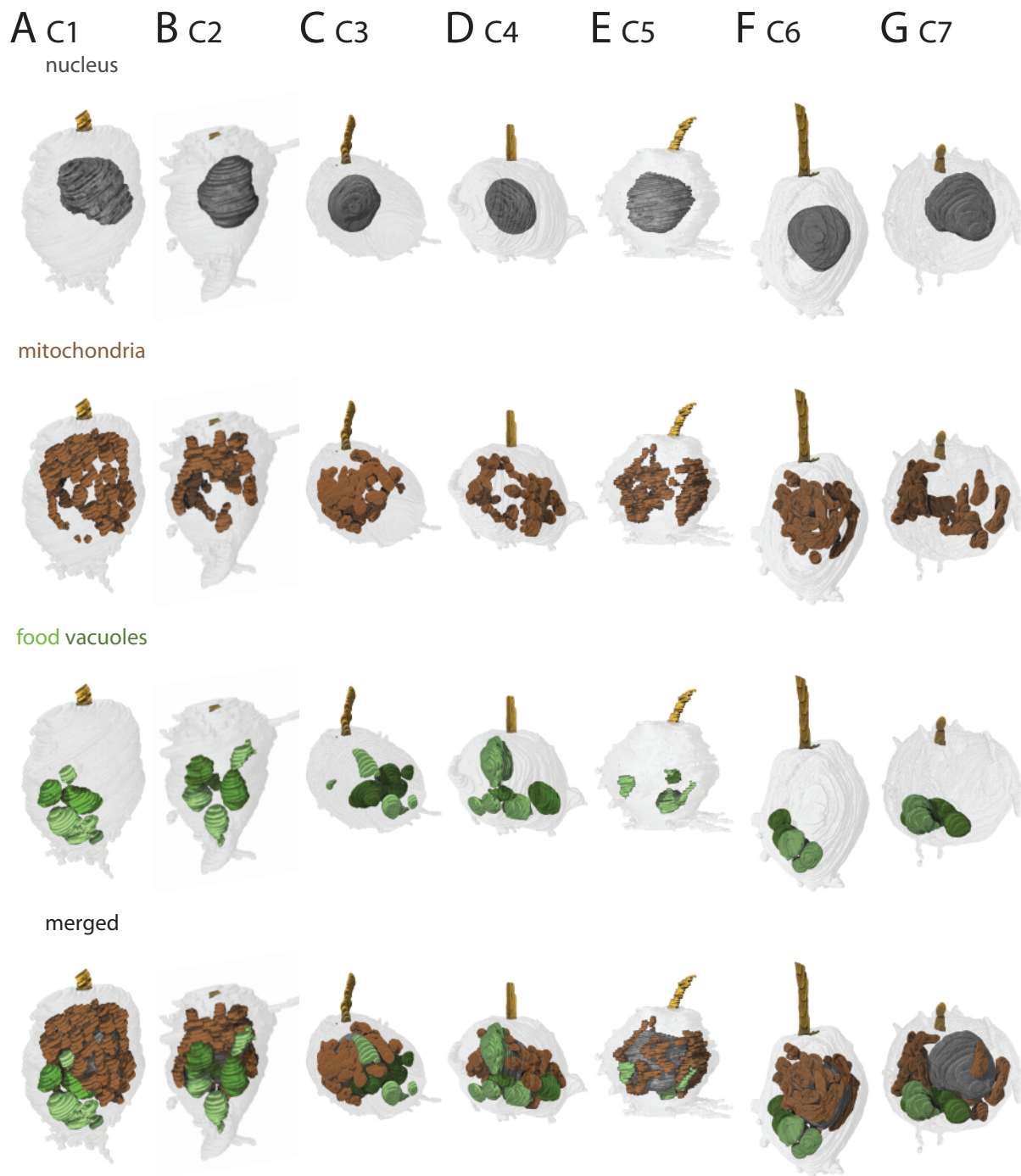

Suppl. Figure 6

Supplement: FIGURE S6 — 3D volume renderings of the nucleus, mitochondrial reticulum, and food vacuoles of cells of a rosette colony of S. rosetta (RC1). (A–G) Cell one (C1) to cell seven (C7). Cells are not to scale. Cells are oriented along the apical (flagellar)–basal axis. The cell body is shown half transparent. The nucleus is colored in dark gray and the mitochondrial reticulum in brown. Food vacuoles with high electron density are colored in light green while food vacuoles with lower electron density are colored in dark gray. [file Image_6.pdf]

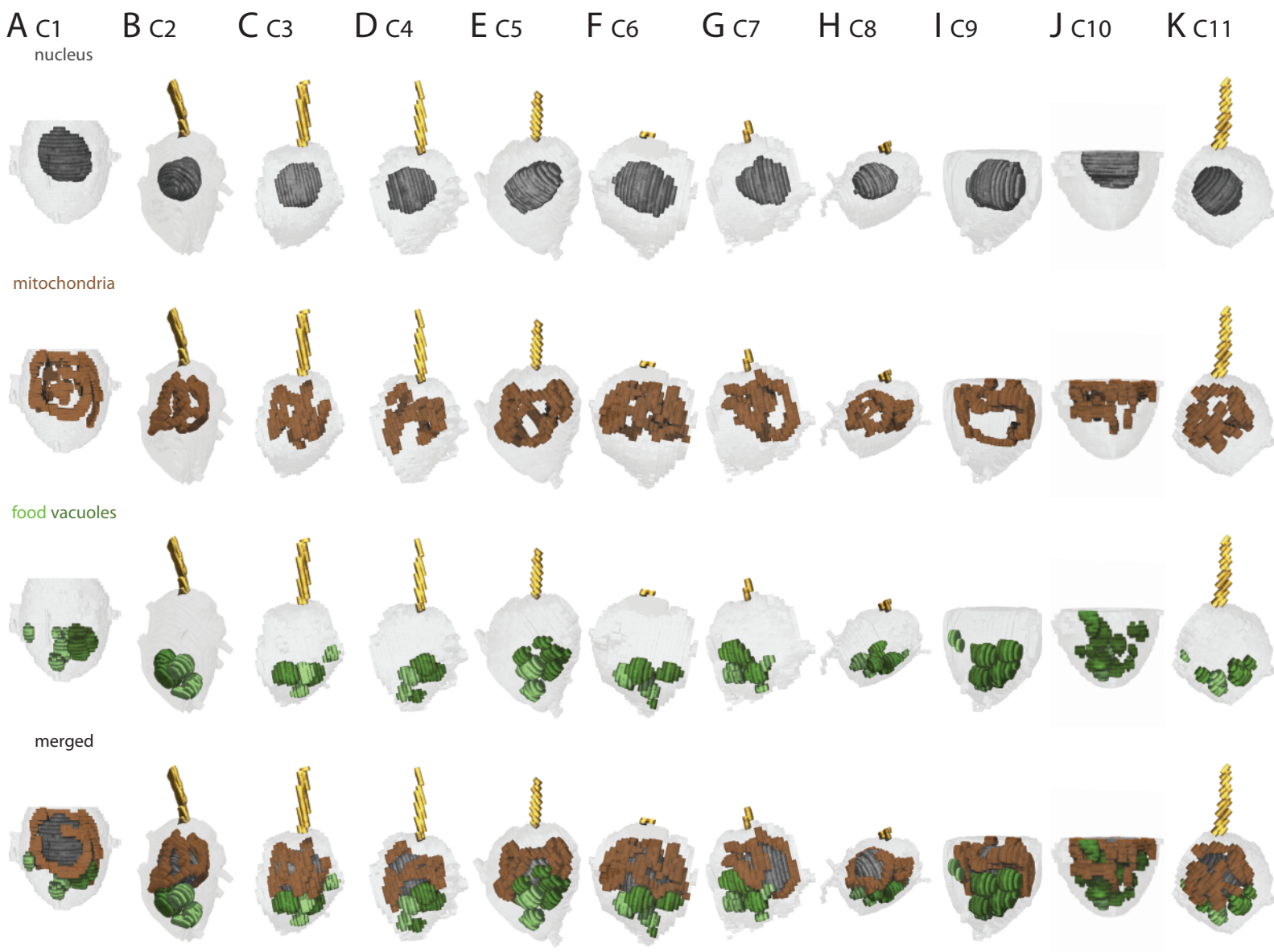

Suppl. Figure 7

Supplement: FIGURE S7 — 3D volume renderings of the nucleus, mitochondrial reticulum, and food vacuoles of cells of a rosette colony of S. rosetta (RC2). (A–K) Cell one (C1) to cell 11 (C11). Cells are not to scale. Cells are oriented along the apical (flagellar)–basal axis. The cell body is shown half transparent. The nucleus is colored in dark gray and the mitochondrial reticulum in brown. Food vacuoles with high electron density are colored in light green while food vacuoles with lower electron density are colored in dark gray. [file Image_7.pdf]

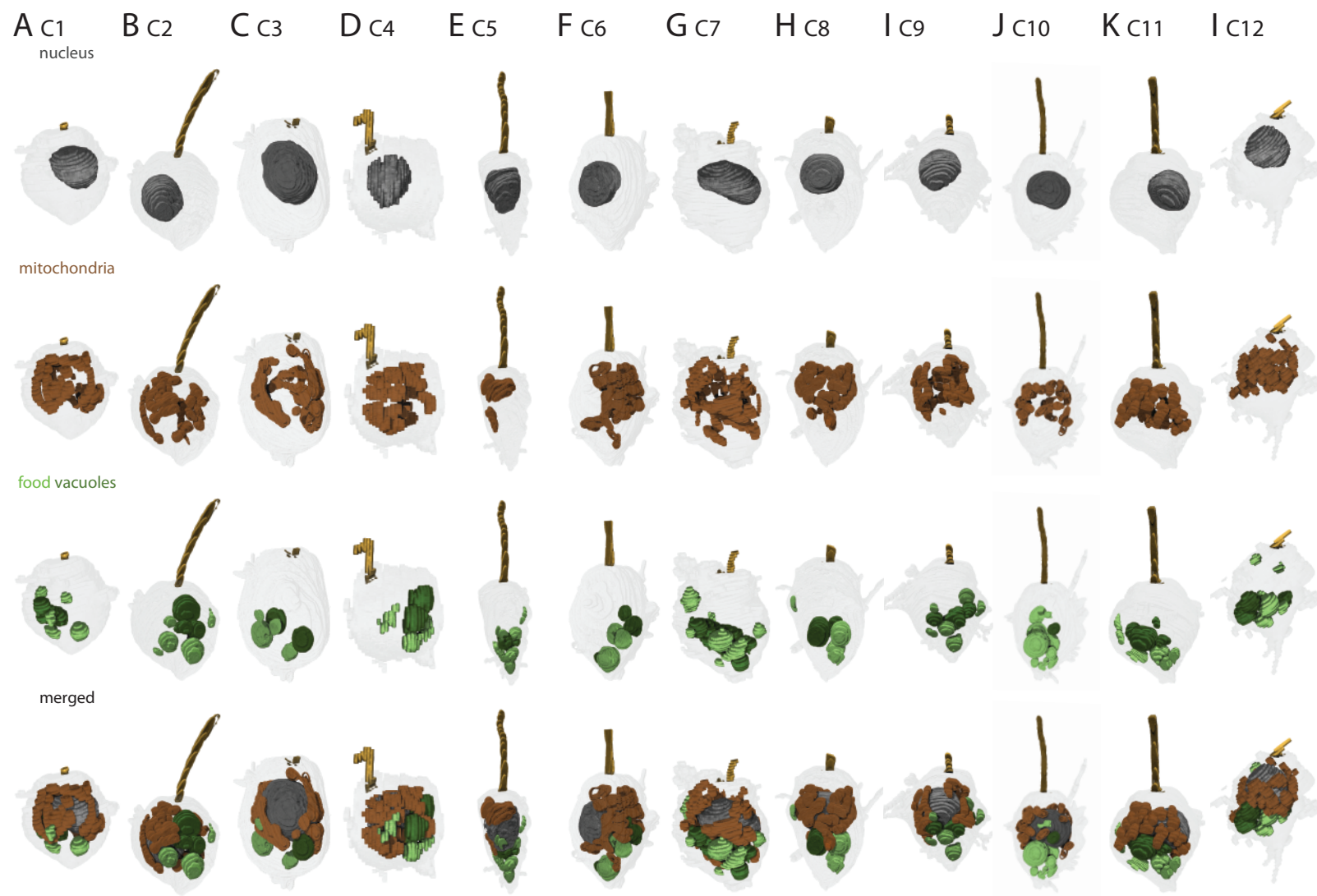

Suppl. Figure 8

Supplement: FIGURE S8 — 3D volume renderings of the nucleus, mitochondrial reticulum, and food vacuoles of cells of a rosette colony of S. rosetta (RC3). (A–L) Cell one (C1) to cell 12 (C12). Cells are not to scale. Cells are oriented along the apical (flagellar)–basal axis. The cell body is shown half transparent. The nucleus is colored in dark gray and the mitochondrial reticulum in brown. Food vacuoles with high electron density are colored in light green while food vacuoles with lower electron density are colored in dark gray. [file Image_8.pdf]

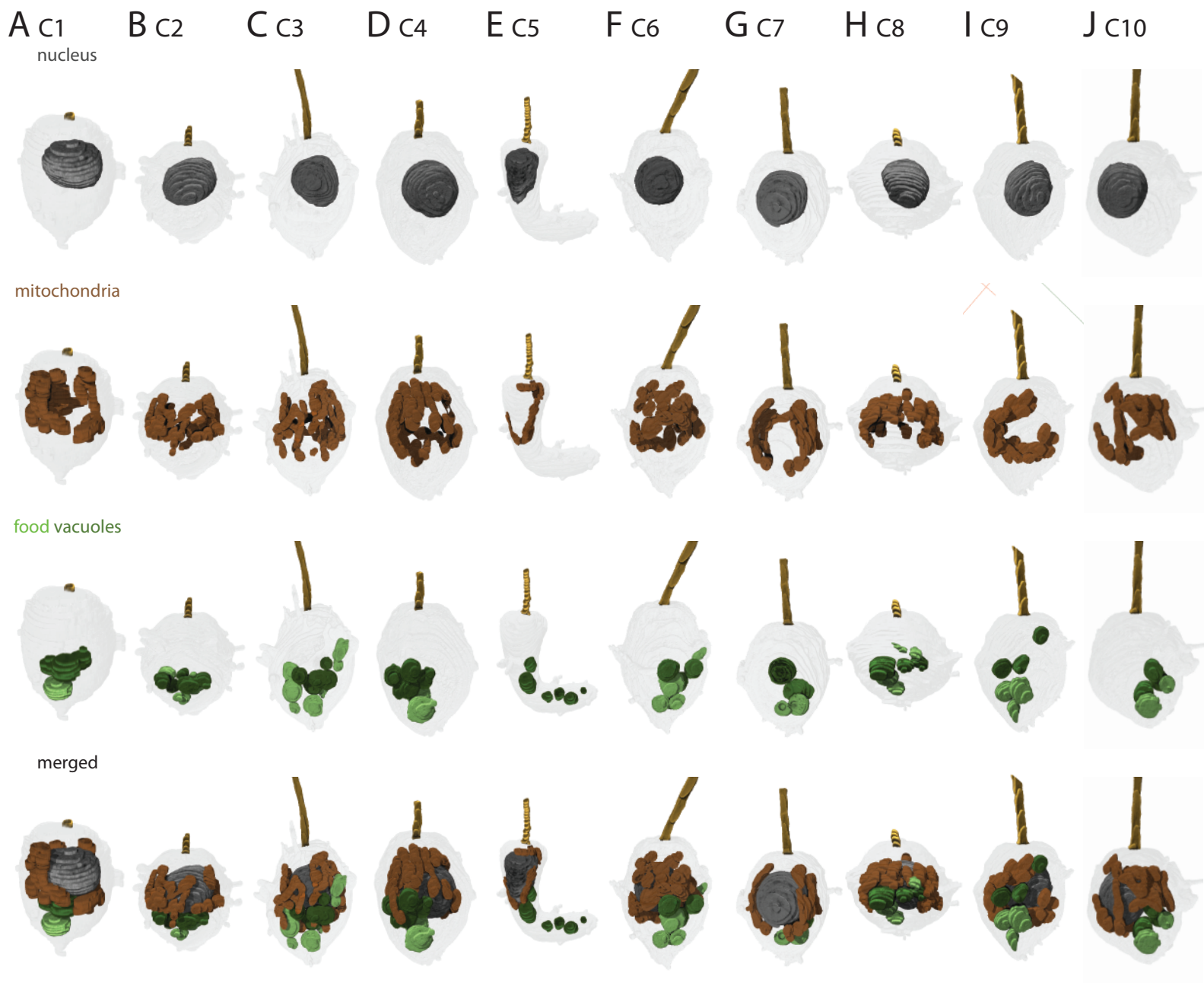

Suppl. Figure 9

Supplement: FIGURE S9 — 3D volume renderings of the nucleus, mitochondrial reticulum, and food vacuoles of cells of a rosette colony of S. rosetta (RC4). (A–J) Cell one (C1) to cell ten (C10). Cells are not to scale. Cells are oriented along the apical (flagellar)–basal axis. The cell body is shown half transparent. The nucleus is colored in dark gray and the mitochondrial reticulum in brown. Food vacuoles with high electron density are colored in light green while food vacuoles with lower electron density are colored in dark gray. [file Image_9.pdf]
