# Supplementary material for: Spatial Cell Disparity in the Colonial Choanoflagellate Salpingoeca rosetta
Source: Front Cell Dev Biol. 2019 Oct 15;7:231. doi: 10.3389/fcell.2019.00231 (PMC6803389; doi:10.3389/fcell.2019.00231)
Supplement: FIGURE S5 — 3D-surface-renderings of cells of a rosette colony of S. rosetta (RC4). Cells are not to scale. (A) 3D-view of the whole colony from different angles. The color spectrum indicates the identity of the different cells. (B–K) Single views of cells of the colony. Cells are oriented along the apical (flagellar)–basal axis. The volume of the whole cell body is given beneath every cell. [file Image_5.pdf]

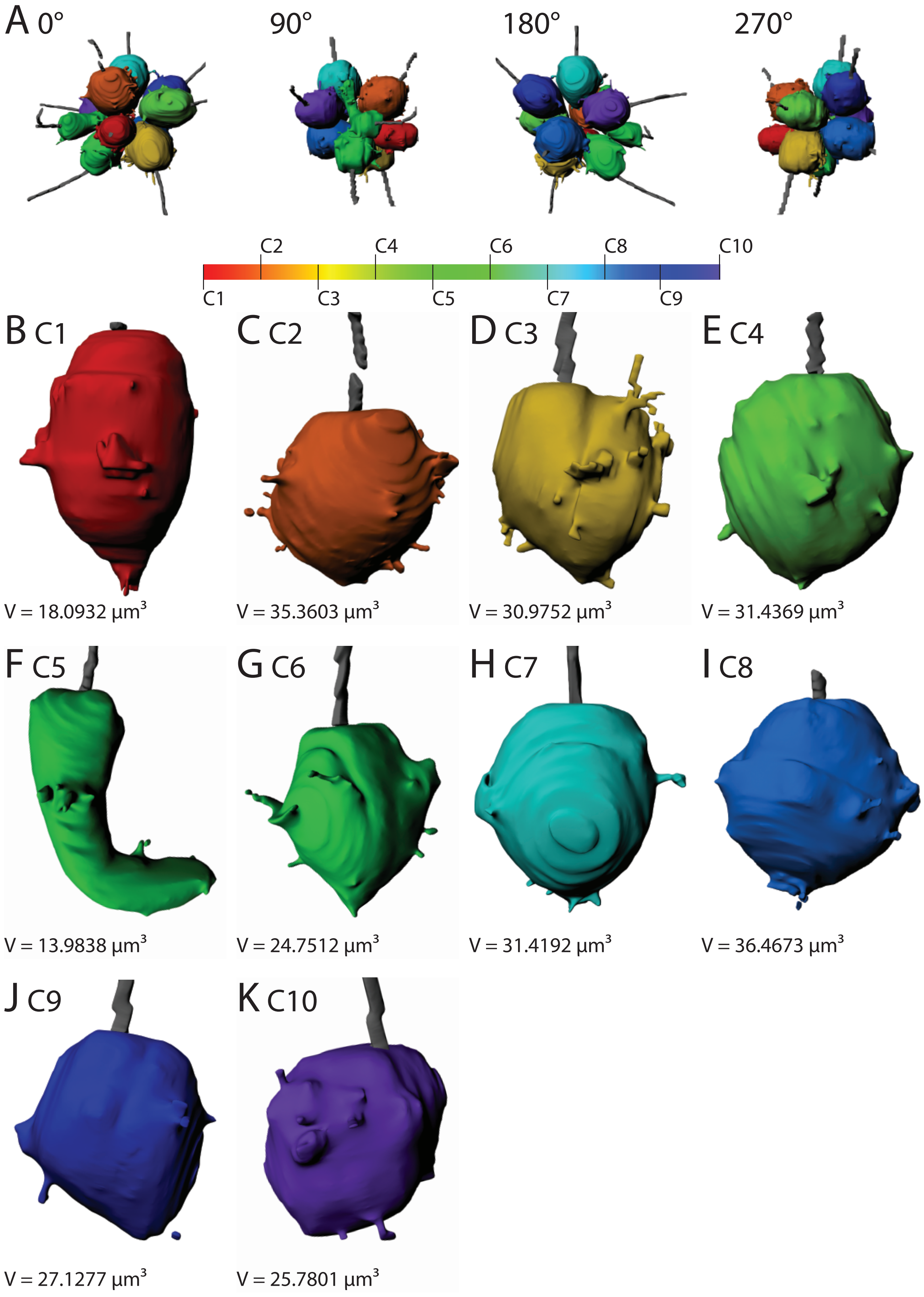

Suppl. Figure 5
